# Supplementary material for: Assessment of the efficacy and safety of two albendazole regimens for the treatment of hypermicrofilaraemic loiasis in adults in Woleu-Ntem Province, Gabon: A phase IIb single-blind randomised controlled trial
Source: PLoS Negl Trop Dis. 2026 Jan 20;20(1):e0013166. doi: 10.1371/journal.pntd.0013166 (PMC12818738; doi:10.1371/journal.pntd.0013166)
Supplement: Attachment — Submitted filename: Responses to reviewers.docx [file pntd.0013166.s001.docx]

**RESPONSES TO REVIEWERS**

**Authors responded in red to comments of editor and reviewers.**

**Reviewers' Comments:**

Reviewer's Responses to Questions

**Key Review Criteria Required for Acceptance?**

As you describe the new analyses required for acceptance, please consider the following:

**Methods**

-Are the objectives of the study clearly articulated with a clear testable hypothesis stated?

-Is the study design appropriate to address the stated objectives?

-Is the population clearly described and appropriate for the hypothesis being tested?

-Is the sample size sufficient to ensure adequate power to address the hypothesis being tested?

-Were correct statistical analysis used to support conclusions?

-Are there concerns about ethical or regulatory requirements being met?

Reviewer #1: The trial includes three groups of individuals: two groups of subjects whose L. loa microfilaraemia was ≥8000 microfilariae per mL (one treated with 400 mg albendazole daily for 30 days, the other treated with 800 mg albendazole daily for 30 days); and a so-called control group, including individuals with a microfilaraemia <8000 mf/mL who received 400 mg albendazole daily for 30 days.

Can we really consider this third group as a "control group"? Yes we can consider this group as a control group.

Why didn't the authors use a placebo group in their trial? Because on the ethical view, it is not acceptable to not treat a patient when it is known he has a disease. Some clinical signs are associated with loiasis even when the microfilaremia is low (inferior to 8000 mf/mL) (Bouyou Akotet et *al*., 2016; M’Bondoukwé et *al*., 2024). Also according to the experience of the Department of Parasitology-Mycology, the ALB-based regimen 400 mg for 30 days is effective and safe. On this basis, patients treated with this regimen fill all criteria to be considered as control group (https://www.ema.europa.eu/en/documents/scientific-guideline/ich-e-10-choice-control-group-clinical-trials-step-5_en.pdf). Moreover when we discussed at the beginning of the study with all local populations, they inform us that they will participate if they will benefit of a treatment against loiasis, intestinal parasitosis and/or malaria.

The methods used to assess the microfilaraemia at the various stages of the trial should be clarified. In the sub-section called "Data collection and screening", it is said that "a 4 mL venous blood sample was collected in an EDTA tube for parasitological screening of L. loa via direct examination of 10 μL of blood". Thank you. We added the following sentence lines 66-67: and leukoconcentration technique.

Was it necessary to collect venous blood at this stage of screening ? Why didn't the authors examine a larger volume of blood (50 µL) taken from the fingertip either by direct examination of the wet smear, or by examination of stained thick smears? Was the leucoconcentration method systematically used during this screening stage for all the individuals? Yes, it was necessary. We collect 4 mL of blood sample for performing the direct examination of 10 µL in a first time to determine the microfilaremia and in a second time, the leukoconcentration technique for improving sensitivity allowing to detect *Loa loa* infection with low microfilaremia (inferior to 100 mf/mL). Both techniques were used at each visit of patients of the clinical trial. We perform the study also in collaboration with the National Parasitic Diseases Control Programme for the establishment of the mapping of loiasis in Gabon. These patients with low microfilaremia represent more than 60.0% of the infected individuals by Loa loa (Akue et al., 2011). And we have for objective to inform the policymakers that loiasis is a problem of public health in Gabon. Low microfilaremia and even patients with no microfilaremia but infected, present symptoms as pruritus, Calabar swelling, subcutaneous migration (Bouyou AKotet et al., 2015; 2016).

The most interesting part of this trial is the comparison of the effects of 400 and 800 mg regimens in the hypermicrofilaraemic subjects. For each timepoint (D2, D7, D14 and D30), the authors should compare the distribution of the individual reduction rates (from D0), using nonparametric Wilcoxon rank-sum tests (Mann-Whitney U test).

Thank you for this contribution. We only performed this analysis at D30. As requested, we performed nonparametric Wilcoxon rank-sum Mann-Whitney U tests comparing the distributions of individual microfilarial reduction rates between the 400 mg and 800 mg arms at D2, D7, D14 and D30. We found that there is no significant difference between reduction rate between in both groups in ITT and PP (p>0.05). We updated the manuscript. Please see the lines 322 to 329.

“**Comparison of the reduction rate median among hypermicrofilaremic patients according to albendazole regimen 400 mg *versus* 800 mg and to intention-to-treat and per-protocol analyses**

Between-arm comparison showed no statistically significant difference at any time point (D2, D7, D14 or D30), both in ITT and PP analyses (p>0.05) (Table 3).

**Table 3**. Comparison of the reduction rate median % at each time point with D0

|  | **Intention-to-treat** | | | **Per-protocol** | | |
| --- | --- | --- | --- | --- | --- | --- |
|  | **Reduction rate median % (95% Confidence interval)** | | | **Reduction rate median %% (95% Confidence interval)** | | |
| **Time points** | **ALB 400 mg** | **ALB 800 mg** | **p-value** | **ALB 400 mg** | **ALB 800 mg** | **p-value** |
| **D2** | 14.0 (-14.1 – 36.0) | 40.9 (-14.9 – 69.2) | 0.4 | 12.5 (-18.9 – 35.6) | 51.5 (-47.0 – 84.0) | 0.7 |
| **D7** | 57.7 (39.0 – 70.7) | 63.4 (45.7 – 75.3) | 0.7 | 43.3 (35.1 – 69.8) | 54.5 (39.7 – 81.4) | 0.4 |
| **D14** | 78.6 (62.0 – 89.1) | 80.6 (64.7 – 89.3) | 0.8 | 79.7 (62.0 – 89.1) | 80.4 (51.5 – 90.4) | 0.9 |
| **D30** | 75.8 (60.7 – 86.6) | 89.6 (78.7 – 93.3) | 0.1 | 77.0 (60.7 – 86.6) | 86.9 (70.3 – 94.3) | 0.3 |

“

**Reviewer #2:** In the introduction, the authors mention the need to find a way to decrease Loa loa microfilaremia in individuals who fail Test and Treat. However, the threshold used to exclude individuals from ivermectin treatment in the Test and Treat studies was 20,000 mf/mL. Why did the authors select 8,000 mf/mL as the threshold for their study since DEC is not used in areas endemic for onchocerciasis. At a minimum, analyses should be included using the 20,000 mf/mL cutoff.

We selected the 8,000 mf/mL threshold based on the findings of Gardon et al. (1997), who demonstrated that the risk of severe adverse reactions after treatment with microfilaricidal drugs increases markedly above this level. Although the Test and Treat studies applied a 20,000 mf/mL exclusion threshold for ivermectin, no clear safety threshold has ever been defined for albendazole, since only isolated case reports of severe adverse events have been published. In this context, we considered the 8,000 mf/mL cut-off to be a conservative and precautionary choice to ensure participant safety.

The reason for the particular choice of control group needs to be made more clear. Presumably, the use of a placebo-control was not acceptable to the population. However, the added benefit of the control group is unclear. An untreated group of patients with high microfilaremia would have been more appropriate. On the ethical view, it is not acceptable to not treat a patient when it is known he has a disease. Some clinical signs are associated with loiasis even when the microfilaremia is low (inferior to 8000 mf/mL) (Gardon et al 1997; Bouyou Akotet et *al*., 2016; M’Bondoukwé et *al*., 2024). Also based on the experience of the Department of Parasitology-Mycology, the ALB-based regimen 400 mg for 30 days is effective and safe. On this basis, patients treated with this regimen fill all criteria to be considered as control group (https://www.ema.europa.eu/en/documents/scientific-guideline/ich-e-10-choice-control-group-clinical-trials-step-5_en.pdf). Moreover when we discussed at the beginning of the study with local populations, they inform us that they will participate if they will benefit of a treatment against loiasis, intestinal parasitosis and/or malaria. So, all tested positive individuals received a treatment.

Why was cetirizine given for 7 days to the control group and albendazole 800 mg? This is not standard concomitant therapy for albendazole, and the antiparasitic effect of albendazole in loiasis takes weeks (as supported by the fact that the patients in this study developed pruritus after 30 days of treatment). Was this also given to the albendazole 400 mg treatment group and, if not, why not, as this would preclude comparison between the groups? Thanks for this observation. Yes three groups received cetirizine for 7 days. We added this information to the lines 161 to 163 in the manuscript : “Moreover, they received a 10 mg cetirizine tablet daily for 7 days. This procedure was carried out under the supervision of a designated community worker within the village to ensure adequate follow-up.”

**Reviewer #3:** yes

**Results**

-Does the analysis presented match the analysis plan?

-Are the results clearly and completely presented?

-Are the figures (Tables, Images) of sufficient quality for clarity?

Reviewer #1: The results of the nonparametric tests should be added in the Results subsection called "Reduction in Microfilaraemia in Intention-to-Treat and Per-Protocol Analyses". Figures 3A and 3B should be completed with new Figures 3C and 3D showing the changes in the MEDIANS (and interquartile ranges) of the microfilarial densities at each timepoint. Done. We added new figures 4C and 4D as requested.

Reviewer #2: The actual time points are not provided in the text or Figure 2. Please see below the modified figure 3 now. I added time points on the left

There are time points in the legends to Figure 5 and 6 but the bars are distributed across the axis, so it is not entirely clear what the actual time point and window were. Thank you for your comments. Please see below the modified figure 6 and 7. Due to revise figure 5 became figure 6 and figure 6, the figure 7.

Please see the figure 6

And the figure 7.

A figure showing the study design would help. Thank you for the suggestion. Please see below the proposed figure 2 corresponding to the study design.

 **Fig *2.* Study design.** This figure correspond to different steps ranging from the screening of participants for the clinical trials to different drug administration (in blue) and carried out procedures at each time points (in green)

Were there safety of efficacy time points after the 30 day treatment? As the authors note, the effect of albendazole on microfilaremia typically peaks at 6 weeks and continues for months. No, they weren’t. We talked about the effect of albendazole at 6 weeks because we have the experience of the use of this molecule in Gabon. And during the follow-up of our patients, this what we observed. Thank you.

Were any of the participants co-infected with other filariae (Onchocerca, Mansonella,..)?
None patient had onchocerciasis (clinical-based diagnosis) but three of them had *Mansonella perstans*.

What were the reasons for exclusion of 84% (336/400) of the Loa-positive patients? This seems like a very large number given the exclusion criteria. What is meant by “unvisited” in Figure 2 – again these numbers are significant (25% of the patients in the high dose group were unvisited at the final time point).
Patient recruitment for this clinical trial was particularly challenging due to the health context at the time. The Gabonese population was largely opposed to COVID-19 vaccination, and upon our team's arrival, many assumed our presence was related to vaccination campaigns. Furthermore, although most participants agreed to provide an initial blood sample, culturally, donating blood is symbolically equated with giving one's life. As a result, they were willing to do so once, but not repeatedly over a 30-day period.

Furthermore, several exclusion factors limited recruitment: 21 participants were absent from the village when the medication was administered; 80 refused to continue after screening; 21 were overweight (>90.1 kg); 34 had previously taken an anthelmintic or a medicinal plant with similar properties; and 25 reported not residing permanently in the study area. 42 had chronic diseases such as diabetes, HIV/AIDS, or hypertension; 14 reported hematuria; 28 reported venous problems during blood collection (difficulty finding veins for venipuncture or problems with the required blood volume); 6 were pregnant; 14 were breastfeeding; 34 had a malaria infection; and 17 had anemia. Please see the flow chart below modified:

What is meant by eosinophilia rate in % in Table 1 – the percent of people with eosinophilia or the mean eosinophil % in each group? Assuming the latter, % eosinophil count is not very useful as it can vary substantially depending on the neutrophil count (absolute eosinophil count is a better reflection of eosinophilia). That said, % eosinophil count is not normally distributed and should be provided as geometric mean and range. Would consider adding range to the geometric mean mf/mL as well. Thank you for your comments. Please see below the table 2 modified with values of the geometric mean and 25^th^ and 75^th^ percentiles for eosinophilia and microfilaremia

**Table 2. Descriptive characteristics of treatment group at the inclusion**

| **Variables** | **Control group (N= 38)** | **Albendazole 800 mg (N=16)** | **Albendazole 400 mg (N=16)** | ***p-*value** |
| --- | --- | --- | --- | --- |
| **Male, n (%)** | 14 (36.8) | 4 (25.0) | 5 (31.2) | 0.7 |
| **Age in years,** mean ± SD^1^ | 49.8 ± 12.4 | 52.2 ± 12.4 | 46.7 ± 12.8 | 0.4 |
| **Weight in Kg,** mean ± SD | 64.2 ± 10.4 | 60.2 ± 10.6 | 63.0 ± 10.1 | 0.8 |
| **Height in cm,** mean ± SD | 164.3 ± 8.4 | 167.6 ± 9.3 | 162.4 ± 7.0 | 0.1 |
| **Microfilaraemia in mf/mL,** geometric mean (min**^3^** – max**^4^**) | 2258 (500 - 7700) | 17249 (8600 - 86000) | 19178 (8200 – 105000) | NA^2^ |
| **Eosinophilia rate in %,** geometric mean (min - max) | 20.4 (3.0 – 27.0) | 15.1 (2.0 – 29.0) | 18.7 (3.0 – 27.0) | 0.7 |

**SD^1^** : Standard deviation

**NA^2^** : Not applicable for the three groups.

**min^3^** : minimum

**max^4^** : maximum

In Figure 4, the authors appear to be showing the proportion of participants meeting the 8,000 mf/mL threshold. However, the text talks about the geometric means and probabilities. This is very confusing. The figure 5 now presents Kaplan-Meier survival curves that estimates the probability of remaining individual above the 8000 mf/mL over time. I changed the expression “geometric mean” by “probability” in the text lines 342 to 345. “…. probability of remaining above 8,000 mf/mL decreased progressively over the 30-day of follow-up (Fig 5A and 5B). The probability decreased earlier in the group of patients with ALB 800 mg before 7 days of treatment contrary to the group receiving 400 mg (Fig 5A and 5B).”

In any case, this data is consistent with prior studies of albendazole, including a placebo-controlled study of albendazole 400 mg twice daily for 21 days, which have shown that some but not all patients with high microfilarial levels show decreases below 8,000 mf/mL. Of note, this earlier trial is mentioned in the introduction (ref 7) where it is incorrectly cited to suggest that albendazole acts on microfilariae but is not mentioned in the discussion despite the fact that the regimen used was most closely aligned with the current paper and it had a placebo-controlled phase IIb design. Thank you. Done. We changed and proposed the following sentence below lines 79-82.

“Previous placebo-controlled studies, such as that of Klion et *al*. reported that albendazole administered for 21 days (400 mg twice daily) led to partial reductions in *L. loa* microfilaraemia in some, but not all, hypermicrofilaraemic patients. These findings suggested that albendazole may progressively reduce microfilarial loads and thus help prepare patients for safer subsequent therapy [7].”

In the discussion section, we added the following paragraph as recommended. Lines 493-499 “Our findings are also consistent with those of Klion et al. [7], who conducted a double-blind placebo-controlled Phase IIb trial in Benin using albendazole 400 mg twice daily for 21 days. In that study, as in ours, some but not all patients with high microfilarial densities experienced reductions below the 8,000 mf/mL threshold. These converging results indicate that albendazole, while not uniformly effective in clearing microfilariae, can nevertheless lower microfilarial loads sufficiently to reduce the number of individuals excluded from further treatment. However, they also reinforce the necessity of microfilarial quantification before treatment, since a significant proportion of patients remain above risk thresholds after therapy.” Lines 509-516.

Reviewer #3: yes

**Conclusions**

-Are the conclusions supported by the data presented?

-Are the limitations of analysis clearly described?

-Do the authors discuss how these data can be helpful to advance our understanding of the topic under study?

-Is public health relevance addressed?

Reviewer #1: In the third paragraph of the discussion, the authors simply compare the percentage of people who fell below the 8,000 mf/mL threshold between the two hypermicrofilaraemic groups. Actually, one of the main result of the study is that the microfilarial densities decreased much more rapidly in the group treated with 800 mg than in those treated with 400 mg. This poses a major safety problem. A regimen using 800 mg is probably not appropriate to treat hypermicrofilaraemic subjects because it could lead to more frequent serious adverse events. Please see below sentences added to the discussion according to your comments : lines 453 to 456 “Importantly, our analyses also showed that microfilarial densities decreased more rapidly in the 800 mg group than in the 400 mg group. This accelerated decline may raise safety concerns in hypermicrofilaraemic individuals, particularly with respect to the risk of adverse events. Lines 460-466.

The text in paragraphs 5

Lines 475-477. “Our own results confirm that the decline occurred earlier and more rapidly in the 800 mg group than in the 400 mg group. While this accelerated clearance reflects the strong efficacy of high-dose albendazole, it may also represent a safety concern in hypermicrofilaraemic individuals, who are at greater risk of adverse reactions when parasite loads fall abruptly. This observation emphasises the need to carefully weigh efficacy against safety when considering higher doses of albendazole.”

and 8

lines 534-537 : “However, the rate of microfilarial decline was clearly faster in the 800 mg group, which could translate into a higher risk of adverse events in hypermicrofilaraemic subjects. This suggests that while the 400 mg once-daily regimen is optimal for balancing efficacy and safety, the 800 mg regimen should be used with caution and warrants further evaluation in larger safety-focused studies.”

of the discussion, as well as the abstract,

lines 36-37: “However, the rapid decreasing of the microfilaremia in the Alb 800 mg group can suggest a better susceptibility to severe adverse reactions.”

should be revised accordingly. In addition, the authors should compare the decrease in microfilarial density they observed in their control group between D0 and D14 and the decrease shown by Klion et al. (reference 7) in subjects from Benin who had received the same dose of albendazole (400 mg per day). The decrease seen by Klion et al. seems to be more progressive. What could be the reasons for this? Done. Lines 433 – 440. “In our control group (patients with baseline microfilaraemia < 8,000 mf/mL treated with ALB 400 mg daily for 30 days), the reduction in microfilarial density between Day 0 and Day 14 was already substantial, with median reductions of around 50.0%. By contrast, in the double-blind placebo-controlled trial conducted by **Klion et al. in Benin**, where patients received albendazole 400 mg/day (200 mg twice daily for 21 days), microfilarial densities remained largely unchanged until Day 14, at which point the first significant decreases compared with placebo were observed. This temporal difference suggests that, in our study, parasite clearance under albendazole occurred more rapidly than in the Beninese cohort. Several factors may explain this discrepancy, including possible variations in study design and treatment supervision.”

In paragraph 6 of the discussion, the authors mention the case of post-albendazole encephalopathy described by Métais et al. They could have also mentioned the case reported by Volpicelli et al. (Parasitology International 2020). Done. Lines 503-523

“Métais and colleagues recently reported a severe and ultimately fatal case of Loa loa encephalopathy following albendazole therapy, in which loiasis was the only identified pathology after exclusion of other causes of coma [22]. This striking case highlights the potential for cerebral inflammation and the need for cautious administration of albendazole in hypermicrofilaraemic individuals. Importantly, it shows that although albendazole is generally considered safer than microfilaricidal drugs such as diethylcarbamazine or ivermectin, serious adverse outcomes can still occur.

Additional reports reinforce this concern. Blum et al. described an encephalopathy temporally associated with albendazole therapy in a loiasis patient who did not present with very high microfilarial densities, suggesting that serious neurological complications are not limited to those with extreme parasite burdens. Instead, these events may be triggered by complex immunopathological responses, irrespective of baseline microfilaraemia [Blum 2001]. Likewise, Volpicelli et al. published a case of albendazole-associated encephalopathy during a 21-day treatment course, after exclusion of alternative causes, thereby further supporting a causal relationship and implicating host–parasite inflammatory interactions as a potential mechanism [Volpicelli 2020].

Taken together, these cases underline that albendazole, while promising as a preparatory or alternative therapy, must be used within carefully validated protocols, especially in individuals with high parasite loads. Although our trial and that of Zoleko-Manego et al. support the relative safety and tolerability of albendazole in populations with heavy parasite burdens, the occurrence of severe neurological complications in the literature demonstrates that risk cannot be excluded. These findings strengthen the rationale for cautious dose optimisation, rigorous monitoring, and the development of clear guidelines for the management of loiasis. In this context, our results suggest that the 400 mg once-daily regimen may represent the best compromise, offering similar efficacy to the 800 mg regimen while potentially minimising the risk of abrupt parasite clearance and associated adverse events.”

In paragraph 7 of the discussion, the authors state that "their findings contrast with those of Gobbi and colleagues, in whom 50.0% of patients treated with 400 mg/day of albendazole remained symptomatic after 30 days". This is not very relevant because among those 5 subjects treated with albendazole only in the Gobbi et al. paper, only two were followed up clinically. So these sentences were deleted in the discussion. “At baseline, 15.7% of patients presented with at least one symptom. The most frequently observed were ocular migration of the worm (26.7%) and pruritus (24.1%). Over the course of follow-up, clinical symptoms gradually decreased across all groups. After 30 days, no symptoms were reported in either the 800 mg or the control group. However, in the 400 mg group, 14.3% of patients still reported pruritus, and 7.1% experienced blurred vision. Although symptoms persisted in some patients, they were minor, indicating a favourable clinical response. These findings contrast with those of Gobbi and colleagues, in whom 50.0% of patients treated with 400 mg/day of albendazole remained symptomatic after 30 days [23].”

Reviewer #2: The impracticality and potential for non-compliance of administering 30 days of albendazole need to be addressed in the discussion as this is a limitation to the approach suggested. We added the following sentences in the section limitations of the study: lines 552-554. “Equally, the impracticality of a 30-day albendazole regimen, which may reduce adherence and increase the risk of non-compliance under real-world conditions.”

Albendazole is known to cause hepatotoxicity and bone marrow suppression in some patients at the doses used in this study. Since laboratory testing was not performed either prior to or during the trial, this limitation also needs to be addressed. Thank you. We added lines 554-556 “To finish, no biochemical laboratory testing was performed during the trial knowing that albendazole can cause hepatoxicity and bone marrow suppression in some patients at the dose used in this study”

As concluded in the double-blind placebo controlled trial of albendazole in loiasis (reference 7), the data form the current study indicate that albendazole given for up to a month would potentially reduce the number of people left untreated during a Test and Treat campaign but would not allow MDA without microfilarial quantification. Lines 441-446, we completed the discussion “Consistent with the findings of Klion et al. [7], our data indicate that albendazole administered daily for 30 days can substantially lower microfilarial densities and thereby reduce the number of individuals who would otherwise be excluded from ivermectin treatment under a Test and Treat strategy. However, as in the Beninese trial, the reductions observed in our study were not sufficient to bring all patients below the 8,000 mf/mL safety threshold. Consequently, individual microfilarial quantification remains indispensable before treatment, and this approach cannot be used as a substitute for mass drug administration without prior screening.”

Reviewer #3: yes

**Editorial and Data Presentation Modifications?**

Use this section for editorial suggestions as well as relatively minor modifications of existing data that would enhance clarity. If the only modifications needed are minor and/or editorial, you may wish to recommend “Minor Revision” or “Accept”.

Reviewer #1: - Introduction, paragraph 1, line 1, please replace "Loa (L.) loa" by "Loa loa"done.
- Introduction, para 1, line 7 : I am not sure that references 2 and 3 are appropriate there. We changed for the references 1 and 2 more appropriate for this sentence.
- Introduction, para 1, lines 5-6: "in individuals … fatal outcomes". Please provide the reference of the papers where the numbers (200-fold, 1,000-fold) come from, as well as information on the reference group. Does this group correspond to the amicrofilaraemic individuals? We thank the reviewer for this important observation. The risk estimates cited (200-fold for ≥30,000 mf/mL and 1,000-fold for ≥50,000 mf/mL) are derived from Boussinesq et al., 2003 (*Filaria Journal*, 2 Suppl 1: S4), which analysed the relationship between *Loa loa* microfilarial density and the risk of severe adverse events following ivermectin treatment. In this study, the reference group used for calculating the odds ratios consisted of individuals with *L. loa* microfilaraemia below 8,000 mf/mL (and not the amicrofilaraemic individuals). We have corrected the reference in the Introduction accordingly.

Please see the following paragraph modified at lines 59-61 “Individuals with microfilaraemia exceeding 30,000 mf/mL are at up to 200-fold higher risk, and those with more than 50,000 mf/mL at up to 1,000-fold higher risk of developing serious or fatal outcomes, compared with individuals with <8,000 mf/mL.”.

- Introduction, para 1, last line: please spell out PNLMT. Done. PNLMP Programme National de Lutte contre les Maladies Parasitaires lines 64-65.
- Introduction, para 2: please explain what the "alternative non-pharmacological strategies" could be

Lines 68-71 : “Currently, no safe and effective treatment is recommended for hypermicrofilaraemic loiasis. Consequently, patients with such high parasitic densities are often left untreated when alternative non-pharmacological strategies are not available, such as vector control measures (e.g., insecticide spraying, larval habitat management) or physical protection with nets, are not available”

- Introduction, para 2, line 4. By definition, a test and treat strategy is not a mass treatment. Thus replace "mass" by "large-scale". Thank you. Done.

- Introduction, para 2, last line. I am not sure reference 4 is appropriate there. Thank you we changed for this one “Kamgno J, Pion SD, Chesnais CB, Bakalar MH, D'Ambrosio MV, Mackenzie CD, Nana-Djeunga HC, Gounoue-Kamkumo R, Njitchouang GR, Nwane P, Tchatchueng-Mbouga JB, Wanji S, Stolk WA, Fletcher DA, Klion AD, Nutman TB, Boussinesq M. A Test-and-Not-Treat Strategy for Onchocerciasis in Loa loa-Endemic Areas. N Engl J Med. 2017 Nov 23;377(21):2044-2052. doi: 10.1056/NEJMoa1705026. Epub 2017 Nov 8. PMID: 29116890; PMCID: PMC5629452.”reference 3.

- Introduction, para 3, line 5. Please replace reference 5 by another reference giving results on the impact of biannual treatment with albendazole alone on the prevalence of lymphatic filariasis. Thank you : please find the reference 4 : Pion SDS, Chesnais CB, Awaca-Uvon NP, Vlaminck J, Abdou A, Kunyu-Shako B, et al. The impact of four years of semiannual treatments with albendazole alone on lymphatic filariasis and soil-transmitted helminth infections: A community-based study in the Democratic Republic of the Congo. PLoS Negl Trop Dis. 2020;14(6):e0008322. doi:10.1371/journal.pntd.0008322.

- Introduction, para 3, line 8: are the authors sure that albendazole "acts on microfilariae"? Please explain what it means (microfilaricidal?, embryostatic?, embryotoxic?) and provide a reference

Thank you for your comment. We changed this sentence by “Previous placebo-controlled studies, such as that of Klion et *al*. reported that albendazole administered for 21 days (400 mg twice daily) led to partial reductions in *L. loa* microfilaraemia in some, but not all, hypermicrofilaraemic patients. These findings suggested that albendazole may progressively reduce microfilarial loads and thus help prepare patients for safer subsequent therapy [7].”

- Introduction, para 3, line 9: please delete "Recently" (the paper (reference 4) has been published in 2016). Done.
- Introduction, para 3, lines 11-12: what is the controversy? It is not clearly defined if albendazole has an impact on microfilariae but as no references are found currently we preferred delete this sentence.
- Introduction, para 4, lines 3-4: what is the meaning of "to use effective molecules for parasitic diseases in the same geographical areas"? Does this refer to integration of NTD control activities? Please clarify. Yes it does. This is why we used the reference of the NTD Roadmap.
- Introduction, para 4, line 4, please replace "The DPMTM" by "The Department of Parasitology-Mycology-Tropical Medicine (DPMTM) of the Libreville Faculty of Medicine". Done. And delete the details at the first line of the Discussion section. Done. The first line was deleted.
- Introduction, para 4, penultimate line: reference 11 is a preprint posted in August 2024. Why do the authors mention PLoS NTD in the reference? This paper was currently published in Plos NTD. We have actualised the reference in the reference list deleting preprint.

- Methods, Inclusion criteria: replace "weigh" by "weight". Done.
- Methods, Exclusions criteria: what was the interval of time between the selection procedure and the first dose of albendazole? The interval of time was between 12 to 24 hours. But we can’t consider it as exclusion criteria because it was difficult to include patients with high microfilaremia and with all inclusion criteria. Please see the figure 3 of the flow chart. Line 213 :” After the screening, an interval of time between 12 and 24 hours for seeing again the individuals has elapsed.”
- Methods, Study design, Study drugs: add "of a village community worker" after "under direct supervision" ; and delete reference to the village community workers in the next paragraphs Done.
- Methods, Study design, Study drugs: please replace "after a fatty meal" by "15 to 30 minutes after a fatty meal (such as fatty rolls with approximately 15 g of butter)". And delete the information on the fatty meal in the "Patients follow-up" sub-section. Done.
- Methods, Study design, Study drugs: if all the groups have received a course of 10 mg cetirizine tablet daily for 7 days, this could be mentioned either before, or after the three sub-sections describing the albendazole regimens, to avoid redundancy. Thank you. Done.
- Methods, Study design, Safety parameters: please replace "or ocular circulation related to" by "or subconjunctival migration of"; Done. and clarify the difference between "Calabar swelling" and "oedema" (the two conditions are distinguished in Table 2). We added at the footnote of the table the following information.

Calabar swelling^1^: transient, localised, angioedema-type swelling caused by worm migration (specific to loiasis)

Œdema^2^: generalised or persistent swelling, often pitting, linked to systemic complications (non-specific).

- Methods, Study design, Patients follow-up : please mention in this sub-section that microfilaraemia was reassessed on D2, D7, D14 and D30. Please explain whether, once the individuals have been selected for inclusion in the trial (after the screening stage), their microfilaraemia was again measured just before the first albendazole treatment, providing the value at D0.

Lines 220-222“. During these visits, venous blood samples in EDTA tubes were also collected to monitor microfilaraemia at D2, D7, D14 and D30. No reassessment of the microfilaremia was performed due to logistical constraints.”

Please clarify whether efforts were made to examine the participants at the same time (hour, minute) of the day at each follow-up visit, in order to limit the impact of the diurnal periodicity of microfilaraemia. Done. Lines 221 to 223. “Efforts were made to examine the participants at the same time (hour, minute) of the day at each follow-up visit, in order to limit the impact of the diurnal periodicity of microfilaraemia.”

The footnote of Figure 2 (No participants were lost to follow-up, as the study design allowed visits at two non-consecutive time points) is not very clear. Thank you for your helpful comment. We would like to clarify that there were no participants lost to follow-up in our trial. Some individuals were not present at certain scheduled visits and are therefore listed as “unvisited participants.” However, these participants often returned for subsequent visits or were assessed at both the inclusion and the final time points. For this reason, we preferred to use the term “unvisited” rather than “lost to follow-up,” as the latter would imply permanent withdrawal from the study. Please see the modified footnote of the figure “with consent. Some participants were not present at specific visits (“unvisited participants”), but they were not considered lost to follow-up, since the study design allowed flexibility with assessments at non-consecutive time points (e.g., inclusion and end-of-treatment visits).” lines 279 to 282.

- Methods, Statistical analysis: please mention here that the analyses were conducted using both ITT and PP approaches. Done. lines 262 to 265. “The reduction rates median expressed in percentage (95% confidence interval) of each visit (D2, D7, D14 and D30) with D0, calculated according to per-protocol and an intention-to-treat approaches were compared between two treatment arms ALB 400 mg and 800 mg.” and Lines 288-289. “The primary endpoint (reduction of microfilaraemia to below 8000 mf/mL) was analysed on both a per-protocol and an intention-to-treat basis.” And see major comment on the use of nonparametric tests to compare reduction rates. Done. Non parametric Mann Whitney test was used to compare reduction rate median.

- Results, table 1 : in the second line, please explain whether the numbers and percentages correspond to males or to females. Thank you. These percentage correspond to males.
- Results, Figure 4 : please explain what the dotted lines correspond to. Do they show the 95% confidence intervals? Thanks. Lines 378-379, we added “The dotted lines correspond to 95% confidence interval.”
- Results, Figure 5, 6B, 6C and 6D: it would be great if the authors could add vertical lines to separate the groups of histograms at each day of follow-up. Done. Figures were modified accordingly. Please see previous responses to reviewer page 4 of the current document.

- Discussion, para 1, line 10: I am not sure that reference 16 is relevant there. Done. deleted
- Discussion, para 1, lines 10-12: what the authors mean by "these protocols could render the entire population in endemic areas eligible for the eradication of this neglected tropical disease"? Do the authors think that the albendazole courses could be used on a large scale, as they suggest in paragraph 10 of the discussion and in the conclusion? Or would they be applicable only at individual level? No we don’t. albendazole protocols can’t be used on a large scale. Done. We changed by “Given the co-endemicity of loiasis and onchocerciasis in areas such as Gabon, these protocols could render individuals in endemic areas eligible for the eradication of this neglected tropical disease.”
- Discussion, para 4: please clarify "no significant difference". When compared to the value at D0? Lines 495-496 “when compared reduction rate of the microfilaremia between D0 and D30,”
- Discussion, para 9, line 1 : replace "older" by "old" or explain what is the comparison population. Done. We changed by “old”

Reviewer #2: Exclusion criteria are labelled as “non-inclusion”. Reasons for withdrawal are labelled as “exclusion”. This is confusing.

Figure 2 is also confusing. The time points are not provided. “Unvisited participants” are listed and it is stated that no patients were withdrawn from study but it is unclear whether any of the participants missed multiple time points and who was included in the PP vs. ITT analyses. Please see the page 8 for the response to this comment. Participants included in the PP approach are those who were present at all visits from D0 to D30 contrary to ITT analyses for which all participant are taken in the analysis at each time point.

Per the text, Figure 3 shows geometric mean reduction in the three groups. A measure of the data dispersion at each point should be added both to the text and the figure. The figure legend should also indicate what is being shown (i.e., geometric mean).

Red and green should not be used as the two colors in the graphs in Figure 4 as approximately 10% of males are red-green colorblind. Please below the modified figure.

**Fig 4. Evolution of microfilaraemia in the treatment and control groups from Day 0 to Day 30.** Panels A and C show the intention-to-treat analysis, while Panel B and D present the per analysis. Panels A and B present the geometric mean of the microfilaremia with the 95% confidence intervals while the panels C and D represent the medians with 25^th^ and 75^th^ percentiles

Reviewer #3: yes

**Summary and General Comments**

Use this section to provide overall comments, discuss strengths/weaknesses of the study, novelty, significance, general execution and scholarship. You may also include additional comments for the author, including concerns about dual publication, research ethics, or publication ethics. If requesting major revision, please articulate the new experiments that are needed.

Reviewer #1: The manuscript submitted by M'Bondoukwé et al. is certainly very interesting because the trial they conducted compared the efficacy and safety of two albendazole regimens in individuals with fairly high Loa loa microfilarial densities. It deserves to be published in PLoS Neglected Tropical Diseases but a number of points should be addressed. Thank you.

Reviewer #2: In their manuscript, the authors present the results of a randomized trial of 30 days of albendazole in hypermicrofilaremic loiasis. The question being addressed (i.e., what to do with individuals who are excluded form treatment using current approaches), is extremely important and this trial adds additional data to the body of published literature. The major strength of the paper is the inclusion of a relatively large cohort of hypermicrofilaremic patients. The manuscript is generally clear and well-written. Specific comments are provided above. Thank you

Reviewer #3: The authors report a clinical phase IIb trial assessing albendazole for the treatment of hypermicrofilaraemic loiasis in an highly endemic region in Gabon. The treatment of hypermicrofilaraemic loiasis is a much debated but unresolved issue and studies providing evidence for informed treatment decisions are therefore of high importance. The team is among the leading institutions in loiasis research worldwide and the study has been meticulously designed, implemented and analysed. The authors have to be congratulated for their work. Thank you

Main limitations of the study are the overall limited sample size and the short follow up period. These limitations are openly addressed by the authors. The conclusion of the paper is of high importance as it provides for the first time evidence for an optimsed treatment protocol for hypermicrofilaraemic loiasis in an endemic setting. Thank you

Please find my specific comments below:

"People living in Central Africa can carry thousands of these microscopic worms in their blood."
Suggest: People living in Central Africa can carry MILLIONS of these microscopic worms in their blood. (individuals may have thousands of mf per ml blood). Done

"help prepare patients for safer treatment with stronger medications and could improve control of this neglected tropical disease in hard-to-reach communities"
Suggest: safer treatment with curative medications. Done

"SARs are common in nearly 10% of the exposed population"
This does not seem correct to me. Please reconsider. Done. SARs risk probabilities after IVM administration were recently evaluated in Cameroon varying greatly according to the number of microfilariae found in the blood : beyond 8,000 mf/mL, the risk is 0.1% and around of 37.0% beyond 100,000 mf/mL [3]. Lines 66 to 68.

Inclusion/Exclusion criteria: better list them in Table than in the text. Done. Please find below the table. (lines 143-145)

### Inclusion/non-inclusion/exclusion criteria were presented in the table below:

**Table 2. Criteria of inclusion/non-inclusion/exclusion of participants in the trial**

| Criteria | | |
| --- | --- | --- |
| Inclusion | Non-Inclusion | **Exclusion** |
| Age between 18 and 75 years; | Having declared chronic diseases such as HIV/AIDS, acute or chronic hepatitis, cancer, diabetes, chronic heart disease, or renal disease; | Withdrawal of consent to participate in the study; |
| Weight less than 90.1 kg; | Taking anthelmintics such as diethylcarbamazine (DEC), suramin, ivermectin, mebendazole, or albendazole within four (4) weeks prior to screening; | Worsening of symptoms or the appearance of severity criteria related to *L. loa* or another disease; |
| *L. loa* microfilaraemia (≥8,000 mf/mL for the treatment group and <8,000 mf/mL for the comparison group); | Being allergic to benzimidazoles; | An increase in microfilaraemia of more than 50% compared to the initial value. |
| Signing an informed consent form; | Being pregnant or breastfeeding |  |
| Agreement to comply with the study procedures, including blood sample collection; |  |  |
| Residing in or being in the village during the clinical trial period. |  |  |

Sample size calculation: please specify the endpoint that was used. In the section sample size calculation we specify that the primary endpoint was *Loa loa* microfilaremia.

What were the most important non-eligibility reasons as there was a substantial proportion not include in the trial?

please see the figure 2 representing the flow chart with a box (above) where we have cited all criteria for which participants with loiasis were non-included.

Please provide SD for microfilaremia in Table 1. Done. Please see the table below. We have also added the minimum and maximum for the eosinophilia rate.

**Table 2. Descriptive characteristics of treatment group at the inclusion**

| **Variables** | **Control group (N= 38)** | **Albendazole 800 mg (N=16)** | **Albendazole 400 mg (N=16)** | ***p-*value** |
| --- | --- | --- | --- | --- |
| **Male, n (%)** | 14 (36.8) | 4 (25.0) | 5 (31.2) | 0.7 |
| **Age in years,** mean ± SD^1^ | 49.8 ± 12.4 | 52.2 ± 12.4 | 46.7 ± 12.8 | 0.4 |
| **Weight in Kg,** mean ± SD | 64.2 ± 10.4 | 60.2 ± 10.6 | 63.0 ± 10.1 | 0.8 |
| **Height in cm,** mean ± SD | 164.3 ± 8.4 | 167.6 ± 9.3 | 162.4 ± 7.0 | 0.1 |
| **Microfilaraemia in mf/mL,** geometric mean (min**^3^** – max**^4^**) | 2258 (500 - 7700) | 17249 (8600 - 86000) | 19178 (8200 – 105000) | NA^2^ |
| **Eosinophilia rate in %,** geometric mean (min - max) | 20.4 (3.0 – 27.0) | 15.1 (2.0 – 29.0) | 18.7 (3.0 – 27.0) | 0.7 |

**SD^1^** : Standard deviation

**NA^2^** : Not applicable for the three groups.

**min^3^** : minimum

**max^4^** : maximum

It is interesting to see that microfilaraemia increased slightly from day 21 in the ALB 400 group. This was also observed in the referenced trial (Manego et al 2023). It would be of interest if the authors could include this in the discussion. Thank you for this helpful comment. We agree that a slight rebound in microfilaraemia was observed after Day 21 in the ALB 400 mg group in our trial. This pattern is consistent with the findings of Zoleko-Manego et al. (2023), who also reported a modest increase in parasite density after an initial decline during albendazole treatment. We have now added this point to the discussion to highlight that albendazole may induce an early reduction followed by partial recovery of microfilarial levels. This may reflect the drug’s limited macrofilaricidal activity and the capacity of surviving adult worms to repopulate the bloodstream once treatment pressure decreases.

Discussion lines 487-491 : In the ALB 400 mg group we observed a slight increase in microfilaraemia after Day 21, despite the overall downward trend. A similar pattern was reported by Zoleko-Manego and colleagues (2023) in their Gabonese cohort, suggesting that albendazole may not fully suppress repopulation of microfilariae once treatment ceases. This rebound could reflect the persistence of viable adult worms and highlights the need for follow-up studies assessing the durability of albendazole’s effects and the potential requirement for combination or repeated therapy.

"however, the ITT analysis suggested this occurred slightly earlier for the 400 mg group (Day 7)
and later for the 800 mg group (Day 11)."
This does not seem to correspond to the Figure. Please comment. Based on the Kaplan–Meier median survival estimates, 50% of participants in the 400 mg group had microfilaraemia below 8,000 mf/mL by Day 7, while in the 800 mg group this point was reached at Day 11. These values are derived from the statistical estimates of the Kaplan–Meier model rather than a direct visual interpretation of the survival curves. Thus, it is possible that this cannot be observed with the curves of the figure 3.

PLOS authors have the option to publish the peer review history of their article ([what does this mean?](https://journals.plos.org/plosntds/s/editorial-and-peer-review-process#loc-peer-review-history)). If published, this will include your full peer review and any attached files.

If you choose “no”, your identity will remain anonymous but your review may still be made public.

**Do you want your identity to be public for this peer review?** For information about this choice, including consent withdrawal, please see our [Privacy Policy](https://www.plos.org/privacy-policy).

Reviewer #1: No

Reviewer #2: No

Reviewer #3: No

**Figure resubmission:**

While revising your submission, please upload your figure files to the Preflight Analysis and Conversion Engine (PACE) digital diagnostic tool, https://pacev2.apexcovantage.com/. PACE helps ensure that figures meet PLOS requirements. To use PACE, you must first register as a user. Registration is free. Then, login and navigate to the UPLOAD tab, where you will find detailed instructions on how to use the tool. If you encounter any issues or have any questions when using PACE, please email PLOS at figures@plos.org. Please note that Supporting Information files do not need this step. If there are other versions of figure files still present in your submission file inventory at resubmission, please replace them with the PACE-processed versions.

**Reproducibility:**

To enhance the reproducibility of your results, we recommend that authors of applicable studies deposit laboratory protocols in protocols.io, where a protocol can be assigned its own identifier (DOI) such that it can be cited independently in the future. Additionally, PLOS ONE offers an option to publish peer-reviewed clinical study protocols. Read more information on sharing protocols at <https://plos.org/protocols?utm_medium=editorial-email&utm_source=authorletters&utm_campaign=protocols>
