# Supplementary material for: Assessment of the efficacy and safety of two albendazole regimens for the treatment of hypermicrofilaraemic loiasis in adults in Woleu-Ntem Province, Gabon: A phase IIb single-blind randomised controlled trial
Source: PLoS Negl Trop Dis. 2026 Jan 20;20(1):e0013166. doi: 10.1371/journal.pntd.0013166 (PMC12818738; doi:10.1371/journal.pntd.0013166)
Supplement: Attachment — Submitted filename: Response to reviewers 07112025.docx [file pntd.0013166.s002.docx]

**Reviewers' Comments:**

Reviewer's Responses to Questions

**Key Review Criteria Required for Acceptance?**


As you describe the new analyses required for acceptance, please consider the following:


**Methods**


-Are the objectives of the study clearly articulated with a clear testable hypothesis stated?

-Is the study design appropriate to address the stated objectives?

-Is the population clearly described and appropriate for the hypothesis being tested?

-Is the sample size sufficient to ensure adequate power to address the hypothesis being tested?

-Were correct statistical analysis used to support conclusions?

-Are there concerns about ethical or regulatory requirements being met?

Reviewer #1: Non-minor comments on the Introduction and Methods sections:
- Line 55: do the authors really think that a 30-day course of albendazole can be applied "on a large scale"? **No, we don’t. We replaced by “The lower dose worked just as well and was better tolerated, making it more suitable for use in individual-scale treatment campaigns”**.

- Line 74: replace "beyond 8,000 mf/mL, the risk is 0.1% and around of 37.0% beyond 100,000 mf/mL" by "the risk is 0.1% for individuals with 8,000 mf/mL and around 37.0% in those with 100,000 mf/mL". In both cases, delete "beyond" : the values are for a GIVEN microfilarial density.

**Thank you for your valuable contribution. We changed according to your instructions.**

- Lines 87-88: same comment as above. Would long course of albendazole be administered at individual or community level? **Thank you. We added “individuals” in the following sentence.”This molecule could also be used to reduce hypermicrofilaraemia in individuals living in well-defined onchocerciasis foci, prior to mass IVM administration (see line 78).**

- Figure 1.: the green dots, which were clearly visible on the first version of the figure, are no longer visible in the Haut-Ntem division after changing the colors. **We changed the figure. Please see page 7 of the manuscript.**

- Table 2: A clarification is needed for the second and third "exclusion criteria". Does this mean that the course of albendazole would be INTERRUPTED (participant’s withdrawal) if there were a "worsening of symptoms or the appearance of severity criteria related to L. loa or another disease" or an "increase in microfilaraemia of more than 50% compared to the initial value" ? If yes, which I suppose, the word "interruption" should be used somewhere. **Yes. Please for more clarification, we added in the table 1 “Withdrawal and interruption of the treatment”**.

- Line 191: I still think that the word "control" is not appropriate because the results obtained in this group are not compared to those obtained with a similar group having benefitted from another intervention. Please delete "control" here (just write "the group with microfilaraemia below 8,000 mf/mL" and do the same throughout the text**. Thank you for your contribution. We proceed to the changing as proposed. Corrected.**

- Line 390: what does "No reassessment of the microfilaremia was performed due to logistical constraints"? Do the authors mean "after D30"?. **Thank you for your contribution. “due to logistical constraints” was replaced by: “No reassessment of the microfilaremia was performed. They received ivermectin if they were positive at D30”**

- Lines 394-413. The quantification of the microfilarial densities is probably much more accurate after leukoconcentration of 4 mL of blood than after examining 10 µL. Thus, I do not understand why both methods were used in parallel at each time point. In the Results section, which data were used ? Those obtained after leukoconcentration or those obtained after examination of 10 µL? **Leukoconcentration was used in addition to direct examination of 10 µL of blood to detect microfilaraemia below the detection threshold i.e. densities lower than 100 mf/mL. So results presented correspond to the combination of both techniques (caption of the figure 4 “The microfilaraemia results presented correspond to the combination of blood direct examination of 10 µL and leukoconcentration techniques. When direct examinaton is negative for *Loa loa*, the leukoconcentration method was subsequently performed”).**

- Lines 440-442: the sentence is very long and confusing. Please split it in two sentences. Does "the reduction rates median" mean "the median of the reduction rates". **Please find the sentence splitted in two with correction. “The median of the reduction rates (95% confidence intervals) expressed in percentage were calculated for each visit (D2, D7, D14, and D30). These calculations were performed in comparison with baseline (D0), according to the per-protocol and intention-to-treat analyses.”**

Reviewer #2: If I am understanding correctly, the microfilariae were quantified in 10 microliters of blood by counting motile microfilariae. Since many of the patients had very high levels of microfilaremia, this seems impossible to do with any accuracy. For example, the participant with 105,000 mf/mL would have 1050 mf/10 microliters of blood. Saponin lysis would be equally difficult to interpret as this patient would have >400,000 microfilariae on the slide. No information is provided re: how accuracy of the counts was ensured. Since the primary outcome of the study is mf count, this issue is essential to address. At the least, data should be provided on the accuracy and reproducibility of the counting method. **Lines 228 : “For the detection and quantification of blood microfilariae, two complementary methods were employed: direct microscopic examination of 10 µL of fresh peripheral blood and the leukoconcentration technique, following the procedures described by Ho Thi Sang and Petithory (1968). The detection threshold of the direct examination was estimated at approximately 100 microfilariae per millilitre, corresponding to the observation of a single microfilaria in 10 µL of whole blood.**

**During direct microscopy, an average of 30 fields was examined to cover the entire 10 µL blood volume. When more than 10 microfilariae per field were observed (equivalent to approximately 30,000 microfilariae per millilitre), a tenfold (1/10) dilution was performed. In cases of high parasitaemia, successive tenfold dilutions were carried out until roughly 10 microfilariae per field were observed. The microfilarial density was then calculated according to the number of dilutions using the following formula:**

**10n×(100×number of microfilariae)**

**In instances where no microfilariae were detected by direct microscopy, the leukoconcentration technique was performed to enhance diagnostic sensitivity, particularly for samples with low microfilarial densities. Approximately 4 mL of venous blood were processed for leukoconcentration: plasma was separated from the cellular pellet which was examined microscopically for the presence of microfilariae.”**

The reason for the cetirizine is still not addressed and is not standard procedure when administering albendazole. Although it is unlikely that this altered the metabolism of albendazole or the frequency of AEs (which typically occur once the mf begin to decrease), this should be clarified. **Administering cetirizine is included into the standard protocol of loiasis in Gabon due to frequent patients complained of itching the first week of treatment after administration of albendazole. These included also pruritus, and administration of cetirizine helped to alleviate the clinical manifestations.** The subheadings in the description of study drug administration could be reduced to a single sentence, assuming that administration procedures and use of cetirizine were comparable in all of the groups. **please see the modified paragraph of the section “Study drugs”. “The investigational product was Ubigen albendazole 400 mg (UBITHERA PHARMA PVVT. Ltd.). The tablets were administered orally, 15 to 30 minutes after a fatty meal (such as fatty rolls with approximately 15 g of butter), to optimise absorption and efficacy. All participants also received a 10 mg cetirizine tablet daily for seven days. Participants with microfilaraemia below 8,000 mf/mL received a single daily dose of 400 mg albendazole for 30 days, whereas those with microfilaraemia ≥ 8,000 mf/mL received either 400 mg or 800 mg (two 400 mg tablets) once daily for 30 days. All treatments were administered under the supervision of a designated community worker within the village to ensure proper follow-up. The field team consisted of parasitologists, lab technicians and field workers who conducted a three-day training session on standardized clinical examination, symptom description, and sample collection techniques for the other study team members, in order to ensure consistency in data collection.”**

Reviewer #3: yes

**Results**


-Does the analysis presented match the analysis plan?

-Are the results clearly and completely presented?

-Are the figures (Tables, Images) of sufficient quality for clarity?

Reviewer #1: Comments on the Results and Discussion sections:
- Lines 521-523 : the sentence " Regarding the group of participants having received ALB 400 mg, there was equally a decrease in ITT - 3825) mf/mL at D30) and PP approaches (16,950 (12,800 - 24975) mf/mL at D0 to 1,950 (675 – 7,325) mf/mL at D30)" needs to be revised. **Thank you. Please find the revise version “Regarding the group of participants having received ALB 400 mg, there was equally a decrease in ITT (17,000 (10,675 – 26,800) mf/mL at D0 to 1350 (700- 3,825) mf/mL at D30) and PP approaches (16,950 (12,800 – 24,975) mf/mL at D0 to 1,950 (675 – 7,325) mf/mL at D30)**"

- Line 556, replace "superior" by "inferior" or "lower". **Corrected.**

- Line 820, why do the authors mention Day 21 here ? (D21 was not a time point in their trial). **Thank you for this comment. We agree that the mention of Day 21 could be confusing, as it was not an evaluation time point in our study. We have therefore removed it from the text and clarified the statement to indicate that the increase in microfilaraemia occurred “between Day 14 and Day 30”.**

Reviewer #2: The values in Table 2 are very small and likely not normally distributed. They should be reported as median or geometric mean and “eosinophilia rate” should be defined. **In the table 2, age, weight and height presented a normal distribution. This is why they were expressed in mean ± SD. However, for the microfilaremia and eosinophilia, they didn’t follow a normal distribution and were expressed in geometric mean (95% confident interval). We added the section “Determination of the eosinophilia rate**

**Eosinophilia rate was determined from the thin blood smear of 5 µL of blood by calculating the percentage of eosinophil among100 leukocytes counted”.**

Reviewer #3: yes

**Conclusions**


-Are the conclusions supported by the data presented?

-Are the limitations of analysis clearly described?

-Do the authors discuss how these data can be helpful to advance our understanding of the topic under study?

-Is public health relevance addressed?

Reviewer #1: Yes

Reviewer #2: The explanation of the more rapid reduction in mf in the present study compared to the prior study is inadequate. Other studies with albendazole in loiasis, including a large placebo controlled trial in Cameroon (PMID: 26967331) have not shown a rapid decrease in microfilarial levels with 800 mg dosing. It is hard to imagine how study design or monitoring could have led to the observed differences. A more likely explanation would be differences in the patient population (genetic differences in the CYP or rate of alcohol/tobacco use which induce these enzymes) and/or parasite (susceptibility to albendazole) between the study sites. **Thank you for your contribution. However, the study by Kamgno *et al.* did not follow the same design as ours. They administered 800 mg every two months among hypermicrofilaraemic individuals, rather than daily as performed in our trial. Therefore, it is difficult to compare results derived from two studies with different designs. Only the studies by Zoleko-Manego *et al.* and Klion *et al.* share certain methodological similarities with ours. Both reported findings showing similar trends to those observed in the present study: microfilaraemia decreased significantly by the end of albendazole 800 mg daily treatment administered for 21 days (Klion *et al.*) and 35 days (Zoleko-Manego *et al.*).
It would be valuable to measure the pharmacokinetics of albendazole in relation to microfilaraemia to determine the optimal therapeutic concentration. Moreover, genetic differences in cytochrome P450 enzymes or variations in alcohol and tobacco consumption could influence albendazole hydroxylation, thereby modifying its efficacy in reducing microfilaraemia. Finally, differences in microfilarial susceptibility to albendazole may also account for the divergent results observed between studies.**

**We added it in the discussion lines 439-455. “In addition to methodological differences, interindividual and population-level pharmacogenetic variability may partly explain the differences observed across studies. Variations in cytochrome P450 (CYP) enzyme activity, particularly within the CYP2J2 and CYP2C19 subfamilies involved in albendazole hydroxylation, can significantly influence drug bioavailability and systemic exposure [20]. These metabolic pathways determine the conversion of albendazole into its active hydroxylated metabolites and ultimately affect therapeutic efficacy. The activity of these enzymes can vary between individuals due to genetic polymorphisms but may also be modulated by environmental and behavioral factors. For example, alcohol and tobacco consumption are known to induce or inhibit specific CYP450 isoenzymes, thereby altering hepatic metabolism.**

**Recent epidemiological data from Gabon demonstrated that both alcohol and tobacco use are more prevalent in rural than in urban populations (19.4% vs 9.6% and 26.1% vs 6.2%, respectively), particularly among men [21]. Such patterns could contribute to altered CYP450 activity in rural participants, potentially modifying the pharmacokinetics of albendazole and partially explaining inter-regional variations in treatment response.**

**Furthermore, differences in Loa loa strain susceptibility to albendazole cannot be ruled out. Genetic diversity among L. loa populations across Central Africa could affect drug – parasite interactions, particularly in genes coding for β-tubulin, the molecular target of benzimidazole derivatives. These host- and parasite-related factors-metabolic, behavioral, and genetic - may collectively account for the heterogeneity observed across clinical trials conducted in different endemic settings, including ours.”** Conversely, although I appreciate that the authors were responding to a reviewer comment, albendazole does not act directly on microfilariae and even in the 800 mg group did not lead to rapid clearing as is seen with DEC or ivermectin. Consequently, the potential risks of the higher dose now seem overstated. **Thank you for the contribution.**

Reviewer #3: yes

**Editorial and Data Presentation Modifications?**

Use this section for editorial suggestions as well as relatively minor modifications of existing data that would enhance clarity. If the only modifications needed are minor and/or editorial, you may wish to recommend “Minor Revision” or “Accept”.

Reviewer #1: Minor comments
- Everywhere in the text and the tables: please harmonize the thousands separators (with commas, eg 2,258, not 2258). **Corrected.**

- Line 34, replace "microfilariaemia" by "microfilaraemia". **Corrected.**

- Line 39, replace "microfilaremia" by "microfilaraemia" (and harmonize throughout the text. **Corrected.**

- Line 40 : replace "better susceptibility to" by "higher risk of". **Corrected.**

- Line 79, delete "are not available" (these words are already at the end of the sentence). **Corrected.**

- Line 84, replace "lead" by "contribute". **Corrected.**

- Line 124: what does the "interruption of the Mectizan programme" mean?. **Thank you for your question. When Gabon was declared ineligible for Onchocerciasis Control Programme and Ivermectin mass drug administration due to onchocerciasis co-endemicity, the mectizan donation programme by MERCK, the only source of affordable Ivermectin for patients, was immediately discontinued until now.**

- Line 311, replace "they received" by "the participants received". **Sorry, but I didn’t find where I used “they received in the manuscript. May be changes were already applied when I answered to another reviewer.**

- Line 323, delete "with the procedure supervised by the village community worker" (already said above). **I changed this part “study drugs” as recommended by another reviewer. Please find the modified paragraph : “The investigational product was Ubigen albendazole 400 mg (UBITHERA PHARMA PVVT. Ltd.). The tablets were administered orally, 15 to 30 minutes after a fatty meal (such as fatty rolls with approximately 15 g of butter), to optimise absorption and efficacy. All participants also received a 10 mg cetirizine tablet daily for seven days. Participants with microfilaraemia below 8,000 mf/mL received a single daily dose of 400 mg albendazole for 30 days, whereas those with microfilaraemia ≥ 8,000 mf/mL received either 400 mg or 800 mg (two 400 mg tablets) once daily for 30 days. All treatments were administered under the supervision of a designated community worker within the village to ensure proper follow-up. The field team consisted of parasitologists, lab technicians and field workers who conducted a three-day training session on standardized clinical examination, symptom description, and sample collection techniques for the other study team members, in order to ensure consistency in data collection.”**

**”**
- Line 504 : what does "1050-5425" correspond to? Minimum and maximum? Please clarify. **Thank you. I modified the sentence “……., the geometric mean (95% confidence interval) decreased from 2,252 (1,050 – 5,425) mf/mL to ………”. Also, I added in the statistical analysis section.**

- Line 507, add "hypermicrofilaraemic" before "participants" ? **Corrected.**

- Line 512, add "in the geometric means" after "reductions" ? **Corrected.**

- Line 513, what does "(-15.1-58.7)" correspond to ? Minimum and maximum ? **No, this is the 95% confidence interval. I added in the statistical analysis section.**

- Title of Fig. 4, the word "protocol" is missing. **Corrected**

- Line 759, differences in the susceptibility of different "strains" of Loa loa could also be considered.
- Line 782, delete "While" ?
- Lines 811-813, merge the two sentences. **In red correspond the lines in the manuscript that we did not find. So we cannot be able to respond to these points.**

Reviewer #2: “Unvisited” is not a standard term and is confusing (suggest that the team did not try to see them). Would use “Missed visit” instead which is the standard monitoring term and indicates only that the visit did not occur. **Corrected**

Figure 2 legend “correspond” should be “corresponds” , “administration” should be changed to “regimens” and the words “carried out” should be removed. There are also many English errors in the figure itself (and in the text added to the manuscript in general) that should be corrected. **Corrected. Please see the modifications in the figure 2. Page 10 of the manuscript.**

I am completely confused by the following statement added in the section on patient follow-up: “No reassessment of the microfilaremia was performed due to logistical constraints.” This seems to directly contradict the preceding and following sentences which talks about sampling at D2, 7, 14 and 30. **Thank you.** **Please find the new sentences “No reassessment of the microfilaraemia was performed. The participants received ivermectin when they were positive at D30”.**

Reviewer #3: (No Response)

**Summary and General Comments**


Use this section to provide overall comments, discuss strengths/weaknesses of the study, novelty, significance, general execution and scholarship. You may also include additional comments for the author, including concerns about dual publication, research ethics, or publication ethics. If requesting major revision, please articulate the new experiments that are needed.

Reviewer #1: The authors have well addressed the comments made by the reviewers.
The results of this trial are most interesting and should be published as soon as possible.
However, the authors should still make some changes before the manuscript can be definitively be accepted.

Reviewer #2: In their revised manuscript, the authors present the results of a randomized trial of 30 days of
albendazole in hypermicrofilaremic loiasis. The question being addressed (i.e., what to do with
individuals who are excluded form treatment using current approaches), is extremely important and
this trial adds additional data to the body of published literature. The major strength of the paper is the
inclusion of a relatively large cohort of hypermicrofilaremic patients. The manuscript is generally clear
and well-written. Specific comments are provided above.

Reviewer #3: The authors have adequately addressed my comments. The table depicting inclusion and exclusion criteria seems somewhat complicated (having the categories inclusion, non-inclusion, exclusion) - I suggest to simplify this table into "inclusion" and "exclusion" criteria only. **Thank you. We changed the title of the columns 2 and 3 by the following : “non-inclusion” was replaced by “Exclusion” and “Exclusion” by “Withdrawal and interruption of the treatment”**

PLOS authors have the option to publish the peer review history of their article ([what does this mean?](https://track.editorialmanager.com/CL0/https:%2F%2Fjournals.plos.org%2Fplosntds%2Fs%2Feditorial-and-peer-review-process%23loc-peer-review-history/1/010f019a1231a8d5-0035381b-84a0-4e28-9111-75d7a8a12e38-000000/E-hgKDR87EsNMQ8BP2CDYXKT2UBLWyGW21VAkEQLqtQ=234)). If published, this will include your full peer review and any attached files.


If you choose “no”, your identity will remain anonymous but your review may still be made public.


**Do you want your identity to be public for this peer review?** For information about this choice, including consent withdrawal, please see our [Privacy Policy](https://track.editorialmanager.com/CL0/https:%2F%2Fwww.plos.org%2Fprivacy-policy/1/010f019a1231a8d5-0035381b-84a0-4e28-9111-75d7a8a12e38-000000/ZY2w6cbR9noAWd2n8XpXouBcxReSIGbiQ2oW99Eph20=234).

Reviewer #1: No

Reviewer #2: No

Reviewer #3: No
