# Supplementary material for: Assessment of the efficacy and safety of two albendazole regimens for the treatment of hypermicrofilaraemic loiasis in adults in Woleu-Ntem Province, Gabon: A phase IIb single-blind randomised controlled trial
Source: PLoS Negl Trop Dis. 2026 Jan 20;20(1):e0013166. doi: 10.1371/journal.pntd.0013166 (PMC12818738; doi:10.1371/journal.pntd.0013166)
Supplement: Attachment — Submitted filename: Responses to reviewers 26122025.docx [file pntd.0013166.s003.docx]

**Reviewers' comments:**

Reviewer's Responses to Questions

**Key Review Criteria Required for Acceptance?**

As you describe the new analyses required for acceptance, please consider the following:

**Methods**


-Are the objectives of the study clearly articulated with a clear testable hypothesis stated?

-Is the study design appropriate to address the stated objectives?

-Is the population clearly described and appropriate for the hypothesis being tested?

-Is the sample size sufficient to ensure adequate power to address the hypothesis being tested?

-Were correct statistical analysis used to support conclusions?

-Are there concerns about ethical or regulatory requirements being met?

Reviewer #1: OK

Reviewer #2: The change to Table 1 has made it more confusing – withdrawal implies interruption of treatment but not the converse. Were there events that could lead to interruption of treatment but not study withdrawal? If not, the title is OK and could be shortened to “withdrawal or treatment interruption”. If there are differences, these should be indicated or listed in two separate columns (study withdrawal, treatment interruption). No, there were no differences. We modified the title of the column of the table 1 as follow: inclusion, exclusion and withdrawal criterias

The quantification of parasitemia is much clearer now. However, dilution amplifies errors (a single microfilaria difference can lead to a thousand-fold difference in count at a dilution of 1:1000). I realize that this may be impossible at this stage but it would be helpful to know the accuracy of the method used (if two people look at the same blood sample, how much variation is there is the count). Thank you for this important comment. We agree that serial tenfold dilutions can amplify counting error, particularly at high dilution factors (e.g., 1:1000), where a difference of a single microfilaria in the counted volume may translate into a large difference after back-calculation.

At this stage, we did not perform a formal inter-reader reproducibility substudy with duplicate independent counts on the same aliquot for all samples. However, to maximise accuracy and limit variability, microfilaraemia quantification followed a standardised procedure: the same fixed blood volume (10 µL) was examined, fields were counted systematically, and dilutions were performed only when densities exceeded the predefined threshold (>10 microfilariae per field). In addition, readings were performed by trained microscopists using harmonised criteria, and any ambiguous or borderline readings were re-checked by a second reader before final recording.

We have now clarified this limitation in the Methods/Limitations and explicitly acknowledge that the absence of a quantified inter-reader variability estimate is a constraint of the present work. We also indicate that future studies will include replicate readings (assessment of agreement using appropriate metrics such as Bland-Altman analysis and/or intraclass correlation coefficients), particularly for highly diluted samples.

We added at the end of the section “Determination of microfilaraemia in individuals with high parasite densities” (line 252 - ) : “Microscopic examination of each slide, including those prepared after serial dilutions, was performed according to a standardised protocol using a fixed blood volume of 10 µL, systematic field examination, and predefined dilution thresholds. Slides with high microfilaraemia requiring dilution were independently examined by two experienced microscopists, who were blinded to each other’s results. Microfilarial density was expressed as microfilariae per 10 µL of blood (mf/10 µL), and the mean value of the two readings was retained for analysis. When a discrepancy greater than 5 mf/10 µL or a borderline count was observed, the slide was reviewed by a third independent microscopist; in such cases, the final microfilarial density corresponded to the mean of the two closest values.”

**Results**

-Does the analysis presented match the analysis plan?

-Are the results clearly and completely presented?

-Are the figures (Tables, Images) of sufficient quality for clarity?

Reviewer #1: OK

Reviewer #2: Comments have been adequately addressed in revision.

**Conclusions**

-Are the conclusions supported by the data presented?

-Are the limitations of analysis clearly described?

-Do the authors discuss how these data can be helpful to advance our understanding of the topic under study?

-Is public health relevance addressed?

Reviewer #1: OK

Reviewer #2: The last statement in the abstract about the potential for serious adverse events should be removed. Albendazole has no direct microfilaricidal effect (the reason for the adverse effects following ivermectin and DEC). This is supported by the finding in prior studies of albendazole and by the data in this paper (see figure 6 and 7 which effectively show little or no difference in post-treatment symptoms between the groups that did and did not receive albendazole). Although the reduction in microfilaremia is slightly more rapid in this study that in the prior studies, it is still much slower and less complete than what is seen with microfilaricidal drugs, consistent with the mechanism of action on adult worms. The rare cases of encephalopathy in the literature may or may not be due to albendazole (encephalopathy has been described in loiasis in the absence of treatment and the conclusions are based solely on a temporal association). These are given too much weight in the discussion. Thank you. We deleted this sentence in the discussion (lines 545-554) “Métais and colleagues recently reported a severe and ultimately fatal case of *Loa loa* encephalopathy following albendazole therapy, in which loiasis was the only identified pathology after exclusion of other causes of coma [24]. This striking case highlights the potential for cerebral inflammation and the need for cautious administration of albendazole in hypermicrofilaraemic individuals. Importantly, it shows that although albendazole is generally considered safer than microfilaricidal drugs such as diethylcarbamazine or ivermectin, serious adverse outcomes can still occur.

Additional reports reinforce this concern. Blum et *al*. described an encephalopathy temporally associated with albendazole therapy in a loiasis patient who did not present with very high microfilarial densities, suggesting that serious neurological complications are not limited to those with extreme parasite burdens. Instead, these events may be triggered by complex immunopathological responses, irrespective of baseline microfilaraemia [25].”

Prolonged treatment with albendazole is, however, associated with other adverse events such as elevated transaminases, cytopenias, and Stevens-Johnson syndrome that are rare but significant (as mentioned in the discussion). Thank you for your valuable comments. We appreciate the sharing of knowledge. We deleted the last sentence of the Methodology and principal findings section of the abstract as recommended. Moreover we added in the discussion lines 551 to 553 “Although our trial and that of Zoleko-Manego et al. [19] support the relative safety and tolerability of prolonged albendazole in populations with heavy parasite burdens, the occurrence of significant but rare severe neurological complications, cytopenias and Stevens-Johnson syndrome in the literature demonstrate that risk cannot be excluded [25,26,27].”.

**Editorial and Data Presentation Modifications?**

Use this section for editorial suggestions as well as relatively minor modifications of existing data that would enhance clarity. If the only modifications needed are minor and/or editorial, you may wish to recommend “Minor Revision” or “Accept”.

Reviewer #1: OK

Reviewer #2: There are numerous remaining grammar, spelling, typographical errors. Examples:
In Figure 1 the use of red lettering on a green background will prevent the 10% of males who are red-green colorblind form being able to see the letters. Corrected. We changed the green into beige background. Please see below .

**Fig 1*.* Study areas. A:** Map of Africa with Gabon highlighted in green. **B**: Map of Gabon with the Ntem and Haut-Ntem departments highlighted in orange. The yellow marker indicates the location of Libreville, the political capital of Gabon. **C**: Black dots represent the villages where participants were screened and/or recruited. The beige area corresponds to the Ntem department, and the blue area corresponds to the Haut-Ntem department. The red triangles correspond to the capitals of the Ntem and Haut-Ntem departments. Base layers: Natural Earth (public domain;<https://www.naturalearthdata.com/about/terms-of-use/>). Administrative boundaries (province and department levels): SALB / UN OCHA, accessed via HDX: <https://data.humdata.org/dataset/d8d992f3-5374-448f-a3d5-bed7c1925a11/resource/46463ad7-36e0-401d-9608-ef4d360c32fe/download/gabadmbndp21msalb.shp.zip>. Village and chief town coordinates: field GPS data collected by the authors (CC BY 4.0).

Line 255 – “all” and “the” are reversed and there is an extra “-or” at the end of the sentence. Thank you. Line 222: “on the all study procedures” changed into “on all the study procedures”
Line 261 - The statement “no reassessment of microfilaremia was performed” is still confusing. Do the authors mean “no reassessment of microfilaremia was performed after D30” – if so, that is how it should be stated. As written it conflicts with the prior sentence describing the reassessment of microfilaremia. Thank you. Corrected.
Line 267 – microfilaraemia is misspelled (micrrofilraemia). Thank you. Corrected.
Line 273 – the verb (“performed”) is missing. Thank you. Corrected.
Line 290 – there is a period missing. Thank you. Corrected

“Eosinophilia rate” is an incorrect term. This would mean the frequency of individuals with an elevated blood eosinophil count. That is not what is described in the methods. The correct heading would be “quantification of eosinophilia”. Thank you. Corrected (Line 271).

**Summary and General Comments**

Use this section to provide overall comments, discuss strengths/weaknesses of the study, novelty, significance, general execution and scholarship. You may also include additional comments for the author, including concerns about dual publication, research ethics, or publication ethics. If requesting major revision, please articulate the new experiments that are needed.

Reviewer #1: OK

Reviewer #2: The authors have responded to the majority of the reviewers' comments. Remaining issues are outlined above.

PLOS authors have the option to publish the peer review history of their article ([what does this mean?](https://track.editorialmanager.com/CL0/https:%2F%2Fjournals.plos.org%2Fplosntds%2Fs%2Feditorial-and-peer-review-process%23loc-peer-review-history/1/010f019b49ffd3ea-cf2c15b3-8836-4fb7-9860-a8c125b3f54f-000000/xThWJf4DRlewc65CV0FwJJwWiESn_LxE2zxPdDTr0ls=242)). If published, this will include your full peer review and any attached files.

If you choose “no”, your identity will remain anonymous but your review may still be made public.

**Do you want your identity to be public for this peer review?** For information about this choice, including consent withdrawal, please see our [Privacy Policy](https://track.editorialmanager.com/CL0/https:%2F%2Fwww.plos.org%2Fprivacy-policy/1/010f019b49ffd3ea-cf2c15b3-8836-4fb7-9860-a8c125b3f54f-000000/hQDu9q6iWf83fHlJuR5mpfL98Vvv-DlsCudMC55aujg=242).

Reviewer #1: No

Reviewer #2: No

**Figure resubmission:**

While revising your submission, we strongly recommend that you use PLOS’s NAAS tool (<https://ngplosjournals.pagemajik.ai/artanalysis>) to test your figure files. NAAS can convert your figure files to the TIFF file type and meet basic requirements (such as print size, resolution), or provide you with a report on issues that do not meet our requirements and that NAAS cannot fix.

After uploading your figures to PLOS’s NAAS tool - <https://ngplosjournals.pagemajik.ai/artanalysis>, NAAS will process the files provided and display the results in the "Uploaded Files" section of the page as the processing is complete. If the uploaded figures meet our requirements (or NAAS is able to fix the files to meet our requirements), the figure will be marked as "fixed" above. If NAAS is unable to fix the files, a red "failed" label will appear above. When NAAS has confirmed that the figure files meet our requirements, please download the file via the download option, and include these NAAS processed figure files when submitting your revised manuscript.

**Reproducibility:**

To enhance the reproducibility of your results, we recommend that authors of applicable studies deposit laboratory protocols in [protocols.io](http://protocols.io/), where a protocol can be assigned its own identifier (DOI) such that it can be cited independently in the future. Additionally, PLOS ONE offers an option to publish peer-reviewed clinical study protocols. Read more information on sharing protocols at <https://plos.org/protocols?utm_medium=editorial-email&utm_source=authorletters&utm_campaign=protocols>
